# Immune Activities of Polycationic Vectors for Gene Delivery

**DOI:** 10.3389/fphar.2017.00510

**Published:** 2017-08-04

**Authors:** Xiaotian Zhao, Xiaoming Li, Yi Zhao, Yuan Cheng, Yunqi Yang, Zhiwei Fang, Yangmei Xie, Yao Liu, Yinghui Chen, Yuanming Ouyang, Weien Yuan

**Affiliations:** ^1^School of Pharmacy, Shanghai Jiao Tong University Shanghai, China; ^2^Department of Neurology, Jinshan Hospital, Fudan University Shanghai, China; ^3^Department of Cancer Biology, Dana-Farber Cancer Institute Boston, MA, United States; ^4^Shanghai Sixth People's Hospital East Campus, Shanghai University of Medicine and Health Shanghai, China

**Keywords:** polycationic vectors, gene delivery system, immune activities, mechanisms, immunoreactions

## Abstract

Polycationic vectors are used widely in the field of gene delivery, while currently their immune activities *in vivo* are poorly understood. In this comprehensive review, we aim to present an overview of existing mechanisms of adverse immune responses induced by the polycation/gene complexes, which includes the polycations themselves, the gene sequences and the ROS produced by them. These causes can induce pro-inflammatory cytokines, hypersensitivity as well as the activation of toll-like receptors, and finally the immunostimulation occur. In addition, we introduce some different opinions and research results on the immunogenicity of classical polycations such as polylysine (PLL), polyethyleneimine (PEI), polyamidoamine dendrimers (PAMAM), chitosan and gelatin, most of which have immunogenicity and can induce immunoreactions *in vivo*. The methods now used to adjust their immunogenicity are shown in the final part of this review. Nowadays, there is still no accurate conclusion on immunogenicity of polycations, which confuses researchers seriously in *in vivo* test. We conclude that further research is needed in order to skillfully utilize or inhibit the immunogenicity of these polycationic vectors.

## Introduction

The successful establishment of gene delivery system can hardly occur without the involvement of vectors, in which viral vectors and non-viral vectors are included. In most cases, viral vectors are modified from adenovirus, vaccinia virus, herpes virus, etc. (Culver et al., 1992; Yang et al., 1994; Puhlmann and Brown, 2000). The high performance of viral vectors in gene delivery is due to their natural ability to infect host cells and release hereditary materials. Meanwhile, a series of safety problems of viral vectors have already been noticed by researchers (Thomas et al., 2003). Non-viral vectors (Duan et al., 2012; Xiang et al., 2012; Chen et al., 2013, 2014, 2016a,b; Ma et al., 2013; Ge et al., 2014a,b), including both natural and artificial polymers, can pack gene sequences *in vitro*, then get into the cytoplasm, and finally release gene *in vivo* through the mechanism of the proton sponge effect (Kesharwani et al., 2012). Currently representative synthetic non-viral vectors include polylysine (PLL), polyethyleneimine (PEI), polypropyleneimine (PPI) and polyamidoamine dendrimers (PAMAM) (Tang et al., 1996; Zauner et al., 1998; Zou et al., 2000; Cloninger, 2002), while widely used natural non-viral vectors are chitosan and gelatin (Erbacher et al., 1998; Truong-Le et al., 1998). Compared with viral vectors, non-viral polymers are often discovered to have higher safety as well as lower immunogenicity and thus are broadly accepted in the use for efficient gene delivery. In all kinds of non-viral vectors, polycations are the carriers most commonly used. When polycations attempt to interact with target cells *in vivo*, they may also interact with immune cells and activate certain immune pathways. For example, macrophages could phagocytose polycation/gene polyplexes *in vivo*, and relevant adverse immune responses were found by researchers (Zolnik et al., 2010). However, the exact molecular mechanism of adverse effects of polycation/gene polyplexes *in vivo* is still uncertain and more attention should be paid to their immunogenicity and adverse immunoreactions.

## Polycations' immune activities related to the immune system

**Figure 1:**
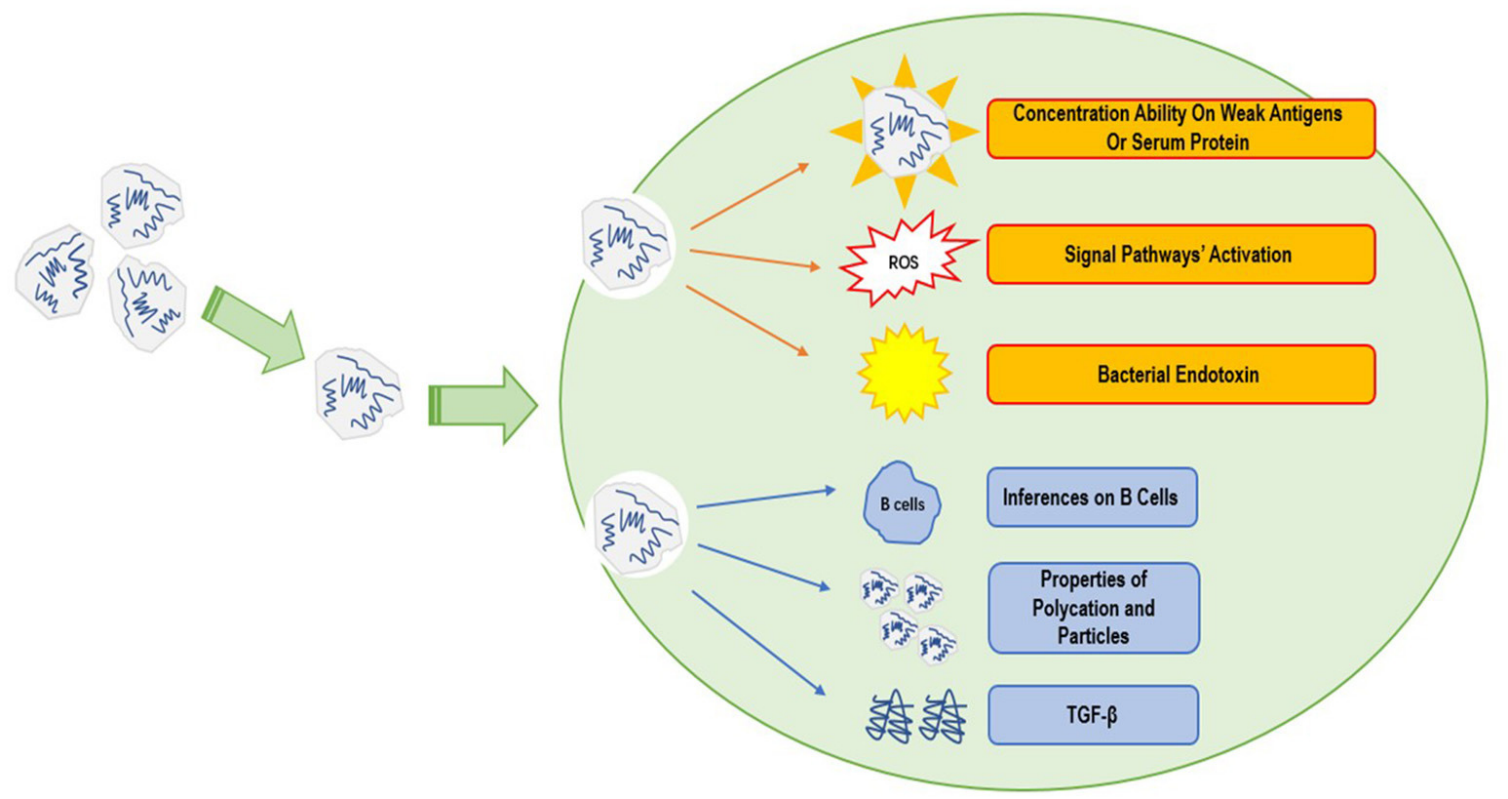
Mechanisms of polycations' immunostimulation and immunosuppression: some polycations' effects on weak antigens, the ROS caused by the positive charge, and the endotoxin sneaked in polycations can stimulate the immune system. Other polycations' effects on B cells and the production of TGF-β can suppress the immune system.

Polycations' effects on the immune system can be generally divided into two categories, which are immunostimulation and immunosuppression (Figure 1). Immunostimulation includes the activation of signaling pathways as well as the induction of antibodies targeting on polycationic complexes. This ability makes the polycations act like immunologic adjuvant (Reddy et al., 2008). It was showed that the immunostimulation had a strong linkage with the particle size of the formed polyplex. In a study by Mottram et al. polrvinyl benzene polyplexes with different particle sizes were used to induce immunological responses on dendritic cells. When particle sizes ranged from 40 to 49 nanometers (nm), immunoreactions generated by poplyplexes were found to be type 1 immunity. When particle sizes ranged from 93 to 101 nm, immunoreactions were type 2 immunity (Mottram et al., 2007). Although the particle sizes tested in this experiment were close to the general sizes of different polyplexes, different polycations may have different immune properties. In addition, with such a narrow range in particle size, it is actually difficult to draw the conclusion about the relationship between immune responses and particle sizes. In another comprehensive study, it was reported that when polycation complexes formed by PAMAM or PPI interacted with serum proteins *in vivo*, those polyplexes could induce the expression of certain antibodies. However, such antibodies could not be found if only polycationic nanoparticles were applied alone, suggesting polycationic polymers alone and polyplexes may have different immune properties (Agashe et al., 2006).

Currently, there is less understanding on immunosuppression compared with immunostimulation. Findings and studies on the relevant molecular mechanisms are also far from enough. The most accepted opinion on the mechanism of immunosuppression is that properties of polyplexes should be the main cause, since the polycations or gene sequences alone could not bring about immunosuppression. As is shown in a study, the formed polyplex could affect the B cells and the production of TGF-β, leading to the immunosuppression (Mitchell et al., 2009). However, another study reported that chitosan was the inhibitor of Type 1 and 2 anaphylaxes (Roy et al., 1999). Therefore, more details and relevant mechanisms of immunosuppression should be further explored in the future.

## Main causes of Polycations' immunogenicity

There are many amino groups on polycationic vectors such as PLL and PEI, which results in their positive charge density *in vivo*. Polyplexes are formed when these positively-charged polymers complex with negatively-charged gene sequences through electrostatic interaction. These polyplexes could bind to proteoglycan or proteins on the surface of the cell membrane, and finally get into the cytoplasm through endocytosis (Ballarín-González and Howard, 2012). Polycations themselves are believed to be able to activate toll-like receptors (TLRs) and then induce the release of cytokines and chemokines such as TNF-α, IL-1β, and IL-6, which would finally lead to the activation of immunoreactions (Zolnik et al., 2010). It was once thought that the main cause of this phenomenon was the positive charge density of polycations (Lv et al., 2006), while a further report stated that even those non-viral vectors without cationic charge could induce immunoreactions as well (Tsukahara and Haniu, 2011).

Furthermore, the gene sequences carried by polycations may also be responsible for the immune responses. If the carried gene sequences code for mRNA, they would be recognized by pattern recognition receptors (PRRs) like TLR-3, TLR-7, and TLR-8, which could result in remarkable immunoreaction; if the gene sequences are plasmid DNA (pDNA), the CpG sequences in pDNA could be recognized by TLR-9. Based on this mechanism, pDNA and mRNA could be seen as immunologic stimulants (Sato et al., 1996; Weide et al., 2008; Tavernier et al., 2011). On account of the inevitable interaction between polyplexes and immunocytes in blood, adverse immune responses will be induced. For instance, macrophages could change into granulomas as a result of hypersensitivity (Poland et al., 2008). This kind of adverse immunoreactions has been reported in previous studies on nano drugs (Dobrovolskaia and McNeil, 2007).

**Figure 2:**
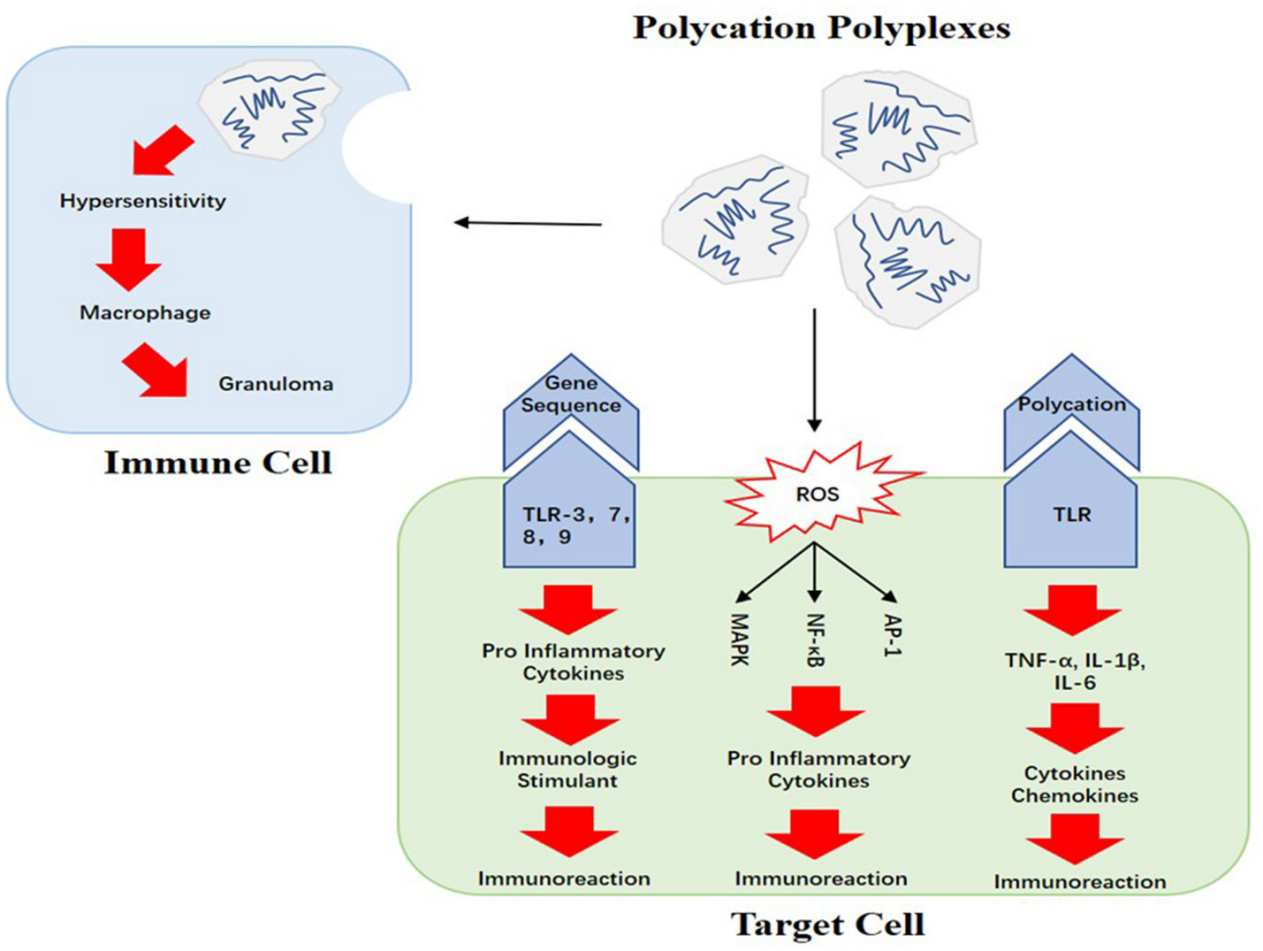
Causes of polycations' immunogenicity: the recognition of carried gene sequences and polycations, ROS produced by polycations, and the impact on immune cells are the main causes of polycations' immunogenicity.

Figure 2 shows the potential causes of polycations' immunogenicity. One study pointed out that when the polycation compounds were in contact with the cell membrane, reactive oxygen species (ROS) such as hydrogen peroxide, super-oxygen ions and hydroxyl radicals could be discovered in those cells. Unfortunately, ROS can activate a series of cellular signaling pathways including AP-1, NF-κB and MAPK (Liu et al., 2012). AP-1 is a type of D-dimer protein, which regulates gene expression when cells are facing certain cytokines, growth factors, bacteria or virus (Hess et al., 2004). NF-κB plays a pivotal role in fighting infections and MAPK is the protein kinase which adjusts cell proliferation, apoptosis as well as differentiation (Pearson et al., 2001; Gilmore, 2006). When those cellular signaling pathways are activated, pro-inflammatory cytokines will be released and immunoreactions such as inflammation will be induced.

There are also other arguments about the potential causes of polycations' immune responses. Some researchers pointed out that it was other factors such as sneaked bacterial endotoxin rather than polyplexes that should be responsible for the adverse immune responses (Vallhov et al., 2006).

## Immune activities of the main polycations

### Chemistry-synthetic polycation vectors

#### Polylysine (PLL)

It was reported in 1975 that PLL could bind to DNA sequences with the help of its electropositivity (Laemmli, 1975), and since then PLL has been utilized extensively as a gene delivery vector. PLL has two kinds of conformation: L and D. Since PLL with L conformation is often found in natural lives, it was more deeply researched and widely applied. Experiments on its immunoreactions were started earlier than the research on other gene delivery vectors. In 1966, PLL (from biopolymer to octamer) was combined with phosphorylated bovine serum albumin to observe its immunoreactions in rabbits. This research found that the pentamer had the most remarkable ability in immunosuppression (Van Vunakis et al., 1966). Further immunological investigations demonstrated that PLL could slightly delay the hypersensitivity (Levine et al., 1968). On the other hand, research on the PLL with D conformation showed that when the terminal of L conformation dendrimer PLL was modified with D conformation, immune responses would be observed and specific IgG could be founded in mice, which actually implied the potential immunogenicity of D conformation PLL (Hudecz et al., 1992). Consistent with this observation, it was also found that pure D conformation PLL can result in obvious immunostimulation. When it was injected into rabbits for the first time, IgG and IgM would be induced. Nonetheless, when it was injected for several times, only IgG could be identified. These antibodies have spatial specificity to D conformation PLL, and immune responses to the L conformation did not occur (Vermeersch and Remon, 1994). Current research show that L conformation PLL is a form of non-viral vectors with none or low immunostimulation and immunosuppression. However, with regard to the D conformation PLL, the problem of strong immunogenicity is still severe.

#### Polyethyleneimine (PEI)

PEI has gradually been regarded as the golden standard among polycations that are used as non-viral vectors for gene delivery after it was first applied in 1995 (Boussif et al., 1995). Due to the existence of plenty of electropositive amino groups, PEI often has excellent ability to deliver gene sequences into cells with the help of the proton sponge mechanism (Behr, 1997). Despite the fact that gene drugs using linear PEI have been researched widely *in vitro*, there are few studies about its immunogenicity *in vivo*. In this case, an immunological research aimed at investigating the immunogenicity of linear PEI (N/P = 8) was performed. Researchers detected some pro-inflammatory cytokines such as IFN-γ, IL-6, IL-12, IL-23 and a series of biological enzymes in the liver, which includes alanine aminotransferase, aspartate transaminase, lactic dehydrogenase as well as alkaline phosphatase. It was found that only IFN-γ was gradually produced by responses to CpG sequences and the judgment was made that linear PEI did not result in significant immunoreactions (Bonnet et al., 2008). In addition, there are other studies pointing out that when DNA sequences with immunogenicity *in vivo* were delivered by PEI, the polyplex could lead to specific immune responses to CD8^+^ T cells (Grant et al., 2012). As a result, much emphasis should be given to the selection of safe gene sequences, even if linear PEI was found to be with no significant immunogenicity.

#### Polyamidoamine dendrimers (PAMAM)

Compared with other polycations, PAMAM have a narrow range of distribution in molecular weight, which implied the easiness in controlling their properties as non-viral vectors (Tomalia et al., 1990). Existing research discovers that PAMAM of G3-G7 have low or none immunogenicity (Roberts et al., 1996). Another study points out that with PEGylation, the immunogenicity of PAMAM can be decreased and the half-time *in vivo* can be elongated (Kobayashi et al., 2001). In a study involving animal experiments, it has been demonstrated that gene drugs utilizing PAMAM did not have severe immunogenicity (Malik et al., 2000). In addition, it was proved that transfection using PAMAM vectors on rabbits' corneas did not result in dangerous immunostimulation (Hudde et al., 1999). However, one study asserted that PAMAM with high molecular weight might be a kind of strong complement activator, while the immunogenicity of low molecular weight PAMAM is relatively inconspicuous (Plank et al., 1996). Owing to this interesting property dependent on molecular weight, PAMAM may have other special use in the field of immunology.

### Natural polycation vectors

#### Chitosan

Chitosan is a type of non-viral vector different from PLL, PEI, PPI, and PAMAM. At most times, chitosan comes from natural plants or animals like crustacea, fungus, or germs. There is a long history of the study on the allergic reactions with crustacea. People now have a general idea about the immunogenicity of chitosan. Chitosan can interact with lytic enzymes and N-acetyl-β-glucosaminidase receptors on the surface of macrophages. Then those macrophages can be activated to release certain non-specific cytokines or other compounds that may play a potentially active role in withstanding bacteria, virus, and tumor (Suzuki, 1982; Nishimura et al., 1986). Chitosan can also induce type Th1 and lower type Th2 immunoreaction *in vivo*. In asthma anaphylaxis mouse models, inhaling chitosan nanoparticles from the nasal area into lung could dramatically lower the immunoreaction, and the asthma symptoms could be alleviated (Shibata et al., 1997). The inhibition of type Th2 immunoreaction was proved in another study (Shibata et al., 2000). One research studying chitosan with different molecular weight found that adverse effects of chitosan were independent of the molecular weight, while the modified chitosan derivatives had increased adverse effects (Kean et al., 2005). Generally speaking, most existing studies on immunoreactions of chitosan are still restricted in the traditional fields like food allergy, and more explorations on the potential mechanisms on molecular and cellular levels should be made in the future.

#### Gelatin

Gelatin has been extensively used in food and drug industry for several decades before it was applied as a gene delivery vector (Bawn, 1987). When the pH value is below 5.0, it can complex with gene sequences and form the polyplex. Much attention is paid to the immunogenicity of gelatin by researchers, since it is a kind of exogenous protein. It has been confirmed that gelatin has low immunogenicity (Schwick and Heide, 1969). Some researchers utilized 60-bloom gelatin as a gene delivery vector and compared its immunoreactions with the liposome. They found that experimental animals had acute immunoreactions when animals were injected with liposome drugs, but similar immunoreactions could not be observed in the gelatin group (Leong et al., 1998). However, there are other researchers holding the position that gelatin nanoparticles can be phagocytosed by macrophages and finally result in the immune response of T cells (Truong-Le et al., 1997). Although people are familiar with gelatin, detailed mechanisms of its immunogenicity are still not fully understood now. The immunoreaction of main polycations have been summarized briefly in Table 1.

**Table 1:** Summary of the immunoreaction of main polycations.

**Immunoreaction of the Main Polycations**	
**Polycation**	**Immunogenicity and inflammatory reaction**
PLL	L conformation: none or low immunostimulation and even immunosuppression; D conformation: strong immunostimulation; (conformation-dependent, IgG, IgM own spatial specificity to D conformation)
PEI	None or low immunogenicity
PAMAM	Low Mw: none or low immunogenicity; High Mw: strong complement activator; (Mw-dependent)
Chitosan	Both immunostimulation and immunosuppression; Activation of macrophages to resist bacteria, virus, and tumor; Inducing type Th1 and lowering type Th2 immunoreaction
Gelatin	Low immunogenicity;Phagocytosed by macrophages; Resulting in immune response of T cells

*PLL, polylysine; PAMAM, polyamidoamine dendrimers; PEI, polyethyleneimine; Mw, molecular weight*.

## Methods of modifying and controlling the immunogenicity of polycations

### Modification of Polycaions' structure

The conventional method used to solve the problem of inherent immunogenicity of polycations is the modification of their structure, in which chemical modification is the most common approach. For example, modifying the two ends or the side chains (Thomas and Klibanov, 2002; Arote et al., 2007; Yang et al., 2015), changing the degree or synthetic methods of polymerization (Kukowska-Latallo et al., 1996; Yu et al., 2016) and modifying the backbone of polycations by adding cross-linking agents (Wang et al., 2002; He et al., 2015; Che et al., 2016; Song et al., 2016, 2017) have been tried to modify the chemical structure of polycations. Modification of chemical structures has been widely used to lower the toxicity of polycations, and actually it did make some excellent achievements. However, we still need to admit the fact that its contributions to the adjustment of immunogenicity are limited.

### Modification of Polyplexes' sizes and surface properties

As mentioned in previous sections of this article, the immunogenicity of polyplexes is dependent on their particle sizes. Thus, better understanding of the specific relationship between sizes and immunoreactions might contribute to the control of immunogenicity. An optimal particle size can be obtained by changing the mass ratio of polycations and gene sequences. However, the number of existing studies on this issue is too small, and the range of ascertainable relationship between sizes and immunoreactions is too narrow to be widely used in future research (Weide et al., 2008). In addition, the great difficulty in establishing well-controlled condition might be a problem as this relationship should be established without the interruption of other potential factors such as charge density and gene difference. Modification of polycations' surface property seems to be a more direct way than changing their particle sizes. Currently existing techniques include PEGylation (Choi et al., 1998; Merdan et al., 2005), preparing diblock or multiblock copolymer (Kim et al., 2007), and linking ligands or antigens with polyplexes, etc. (He et al., 2015).

### Modification of gene sequences

With the growing knowledge in the immunogenicity of gene sequences, some researchers have tried to modify the RNA or DNA sequences delivered by polycations. Some nucleosides in the original mRNA were replaced by modified nucleosides such as 5-methyl-cytidine, 2-thio-uridine, and pseudo-uridine. It was discovered that the immunogenicity of modified mRNA was reduced, while the transfection efficiency, protein expression ability and stability of mRNA changed erratically (Uchida et al., 2015). As a result, this kind of modification method needs further exploration to find a balance between safety, efficiency and stability.

## Discussion

### The immunogenicity of classical polycation vectors remains unclear

Although those classical polycationic vectors have been used for several decades, studies on their immunogenicity are unfortunately at a standstill. Previously, it was even universally believed that non-viral vectors have low or none immunogenicity. However, nowadays, there are different ideas and unconfirmed hypotheses about their immunogenicity as we demonstrated in this article. Another issue is that those basic studies on classical polycationic vectors were done around 20 years ago and there are not enough studies that try to verify the previous hypotheses by using modern technologies.

### New polycationic vectors lack research on immunogenicity

Compared with those classical polycationic vectors, newly developed polycations are often with better gene delivery capacity and fewer adverse reactions. In most cases, we are usually attracted by the outstanding performance of polycations in cell cytotoxicity or transfection efficiency, and tend to ignore the adverse immunoreactions *in vivo*. We believe that more emphasis should be laid on the immunogenicity of newly developed polycations. The progress in this field may guide us to sensibly apply non-viral gene therapy in the future.

### The immunogenicity caused by polycationic vectors can be utilized sensibly

Currently, gene vaccine is an emerging research area in gene therapy. We consider it as an applicable example to prove the use of immunogenicity in gene drugs, though the principle behind gene vaccine is different from that of polycations' immunogenicity. Inspired by the use of gene vaccine, polycationic vectors may also have huge potential as delivery agents of gene vaccines or even antigens stimulating the immune system. Thus, we believe that immunoreactions of polycations should be averted as much as possible when they are used as gene delivery vectors; however, with regard to the potential use in gene vaccine, their immunostimulation ability can be utilized to enhance the immune response.

## Conclusion

The immunogenicity of polycationic vectors mainly includes immunostimulation and immunosuppression. The immunostimulation might be caused by polycations themselves, gene sequences as well as those polyplexes, and the immunosuppression is believed to be caused by the responses of human immune system to polyplexes. The mechanism of immunostimulation has been elucidated in more detail than that of immunosuppression. In order to change and control the immunogenicity of polycationic vectors, different methods have been employed including the modification of the polycation structure and surface characteristics, the adjustment of particle sizes, and the modification of the nucleosides of gene sequences. Great efforts are still needed for future studies on immune activities of polycationic vectors in gene delivery.

## Author contributions

WY conceived the initial idea and the conceptualization, and designed the experiments, participated in the data extraction and analysis, and revised the manuscript. XZ, XL, YZ, YCheng, YY, ZF, YX, YL, YChen, and YO. conceived and participated in its design, searched databases, extracted, and assessed studies and helped to draft the manuscript. XZ wrote the manuscript. YChen, YO, and WY revised the manuscript. All authors read and approved the final manuscript.

### Conflict of interest statement

The authors declare that the research was conducted in the absence of any commercial or financial relationships that could be construed as a potential conflict of interest.
